# Pharmacokinetics of ranitidine in preterm and term neonates with gastroesophageal reflux

**DOI:** 10.1186/s12887-016-0630-x

**Published:** 2016-07-13

**Authors:** Ismael Lares Asseff, Graciela Benitez Gaucin, Hugo Juárez Olguín, Jose Antonio Godinez Nájera, Alejandra Toledo López, Gabriela Pérez Guillé, Fausto Zamura Torres

**Affiliations:** Interdisciplinary Centre of Investigation for Integral and Regional Development, Durango Unit, National Polytechnic Institute, Durango, Mexico; Pediatrics and Neonatology Services, General Hospital of Durango, Durango, Mexico; Laboratory of Pharmacology, National Institute of Pediatrics, and Department of Pharmacology, Faculty of Medicine, National Autonomous University of Mexico, Av Imán #1, 3er piso, Col Cuicuilco, CP 04530 Mexico City, Mexico

**Keywords:** Gestational age, Full-term, Neonates, Pre-term, Ranitidine, Pharmacokinetics

## Abstract

**Background:**

The aim of this study was to determine the effect of gestational age on pharmacokinetics of ranitidine in newborns with gastroesophageal reflux.

**Methods:**

A prospective, descriptive and pharmacokinetic study was carried out in 30 pre-term and 20 full-term babies. 3 mg/kg of ranitidine was administered intravenously to all the babies and at 0.25, 0.5, 1, 2, 4, and 8 h following the administration, samples of blood were drawn to assess ranitidine levels using high performance liquid chromatographic technique.

**Results:**

Pharmacokinetics of ranitidine had a bi-exponential behavior with a half-life elimination of (t_1/2_el) 2.79 h, area under curve (AUC) of 1688 ng/mL, volume of distribution (Vd) of 1.44 L/kg, and clearance (Cl) of 5.9 L/kg/h. The median plasmatic concentration in pre-terms was 1113 ng/mL and 280 ng/mL in full-terms. Vd, t1/2 and Cl presented high values in preterm although the correlation of Cl with glomerular filtration in term newborns was better.

**Conclusions:**

Plasma levels of ranitidine depend on the gestational age of the newborns. However, the possible relationship between after-birth age and pharmacokinetics of the neonates as their internal organs get matured without minding their gestational background.

## Background

Ranitidine decreases gastric acid secretion and improves esophagitis. It is then fundamental to have a good knowledge of its pharmacokinetic activities so as to make the best use of it in the treatment of every patient [1–4]. In normal newborns and infants, several organ systems progressively get matured to culminate in the later life and progressive maturation depends on the organ system and the specific iso-enzyme involved [5, 6]. In a study by Hedenström et al., [7], on pharmacokinetics of ranitidine in adults, it was found that the use of multiple dosing scheme of ranitidine has similar parameters as the use of single dosing scheme. Its concentration, following intravenous administrations fits to a bi-exponential kinetics. In nonates, the half-life elimination is almost 2 h, and it is a little more prolonged after oral administration [8]. Hepatic metabolism is one of the elimination route and this suggests that the drug has enterohepatic re-circulation for bi-exponential rate [9].

Due to the high risk of either overdosing or under-dosing in newborns, which could lead to either therapeutic failure or toxic effect, the study of pharmacokinetics of ranitidine in this group of patients is very important [1, 10, 11]. The absence of specific ranitidine dosing scheme for newborns has cornered physicians to use doses obtained by modifying the dosing schemes for older and mature children. The result of this is usually overdosing or sub-therapeutic dosing scheme.

In a study of pharmacokinetics of ranitidine in 27 full-term newborns without liver or kidney problems using 2.4 mg/kg of ranitidine, Fontana et al., [12], found that the half-life elimination (t1/2el) of the drug was 3.45 ± 0.31 h, the total distribution volume (Vd) was 1.52 ± 0.91 L/kg and the total plasmatic clearance (CL) was 5.02 ± 0.46 mL/kg/h. However, the above study did not consider preterm neonates. Assuming that these measurements do not change with different dosing schemes, they could be used to derive a treatment plan for newborn, since the age-specific pharmacodynamics could only be evaluated after knowing the age-specific pharmacokinetics.

In view of the above findings, the aim of this study was to determine the effect of gestational age on pharmacokinetics of ranitidine in newborns with gastroesophageal reflux to provide guidance on ranitidine therapeutic schemes for this age group.

## Methods

A prospective, descriptive and pharmacokinetic study of 50 newborns (30 males and 20 females) receiving ranitidine at Neonatal Intensive Care Unit (NICU), General Hospital, Durango, Mexico, was carried out with the objective of determining the pharmacokinetics of the drug.

All the patients, by medical prescription, required treatment with ranitidine. To avoid nephrotoxicity, serum creatinine and its renal clearance were determined as an inclusion criterion and once the values were confirmed to be normal, the patients were made to receive a first dose consisting of 3 mg/kg/day of ranitidine by slow intravenous injection (during two min) of multiple doses. A sample of 0.5 mL of whole blood was drawn from neonates at 0.25, 0.5, 1, 2, 4, and 8 h following drug administration, and were separated by centrifugation and stored for no more than 30 days at −80 °C until analysis. However during the validation procedure the samples assayed showed stability for at least 60 days.

During the study intragastric pH was registered by gastric aspirates. The research protocol was approved by the Research Ethics Committee of the General Hospital of Durango, México, and informed consent was obtained from the parent or guardian of each patient, according to principles of Helsinki Declaration of 1975. Acceptance into the study did not involve greater risks than those related to sampling. The volume was minimal and did not put at risk the balance or imbalance of fluids. The sample size was calculated using the formula reported by Castilla and Cravioto [13].

Pre-term and full-term neonates of 0 to 28 days old that required ranitidine treatment with normal liver and kidney functions were included in the study. All patients requiring treatment with ketoconazole or antacids agents were excluded.

### Analytical procedure

Plasma ranitidine concentrations were analyzed using High performance liquid chromatography (HPLC), Agilent 1100 chromatographic system. The method was adapted from a previously published method by Castañeda et al., [14]. Quantification was carried out by using the ratio of area under the curve for ranitidine and nizatidine as standard internal calibration curves, and linearity was assessed in concentrations of 0, 50, 100, 250, 500, 1000 and 1500 ng/mL for solution and plasma. A correlation coefficient of *r* = 0.999 was determined between intra and interday.

Mean accuracy of known concentration ranged from 99.4 to 103.5 %. Recovery was assessed by determining three concentration levels in plasma and solution (75, 600, and 1200 ng/mL). The minimal concentration that could be accurately measured was 15 ng/mL. A coefficient of variation (CV) of less than 7 % was obtained. The percentage of recovery was 108 %, which is within the range recommended by Official Mexican Standard [15]. The response of plasma samples, to which ranitidine had been added at the afore-mentioned concentrations was compared with the response of ranitidine solutions at the same concentrations.

To 200 mcL of plasma, 50 mcL of Nizatidine (internal standard) and 50 mcL of NaOH 2.5 M were added. The mixture was lightly shaken in vortex and then 3 mL of dichloromethane was added to it and shaken again for 1 min in vortex, and centrifuged for 5 min at 3000 rpm. Once centrifuged, the organic phase was separated from the aqueous phase and air-evaporated at a temperature of 45 °C. It was then reconstructed with 200 mcL of mobile phase, and 50 mcL of this mobile phase was injected to the system [14]. A chromatographic symmetry C18 of 5 μm and UV detector with diode arrangement with flow velocity of 1 mL/min at a wavelength of 313 nm was used. For mobile phase, a mixture of monobasic potassium phosphate 0.05 M, pH 6.5 and acetonitrile (88/12, v/v) was used, with an injection volume of 100 mcL. The retention time of ranitidine was 2.82 min, 4.2 for IS and 6.5 as total chromatographic run time.

The pharmacokinetic profile of ranitidine was estimated in plasma concentrations obtained at different times [16]. Non-linear regression was used to fit plasma ranitidine concentrations to a two-compartment (bi-exponential) model [17]. The WinNonlin software (Version 2) [18], was used for all non-linear regression, and to generate all pharmacokinetic parameters. A similar analysis was performed with observations either in preterm or term cases.

## Results

Fifty newborns that were admitted at Neonatology Unit of Hospital General de Durango, Mexico that required therapeutic management with ranitidine were studied. All newborns presented risk factors susceptible to digestive tract bleeding.

The characteristics of the patients based on gestational age were as follows: 6 pre-term babies (babies born before 37 weeks of gestation) and small for their gestational age (SGA) (weight < 10^th^ percentile); 20 pre-terms, appropriate for their gestational age (AGA) (weight between 10 and 90 percentile); 4 pre-terms, large for their gestational age (LGA) (weight > 90 percentile); 7 full-terms (babies born between 37 and 42 weeks), small for their gestational age (SGA); and 13 full-terms, appropriate for their gestational age (AGA). Table 1 shows a comparative analysis of the statistically significant differences between gestational age and birth weight of preterm and term. It can be observed that the values in preterm newborns were much lower (Latin American consensus) [19].

**Table 1:** Demographic data of newborns whose pharmacokinetic of ranitidine were studied

Biological Characteristics	Females	Males	Statistical test	*P* value*	Preterm	Term	P*
	*n* = 20	*n* = 30			*n* = 30	*n* = 20	
Gestational age (weeks) Mean ± SD	34.2 ± 4.16	35.8 ± 2.7	2.76	0.103	33.6 ± 3.7	38.3 ± 0.9	0.0001
Birth weight (g) Mean ± SD	2330.7 ± 677	2680.5 ± 561	0.135	0.715	2361.9 ± 563.0	3000.8 ± 620	0.008

*SD* standard deviation

*Square Chi Test (*X* ^2^)

**Fig. 1:**
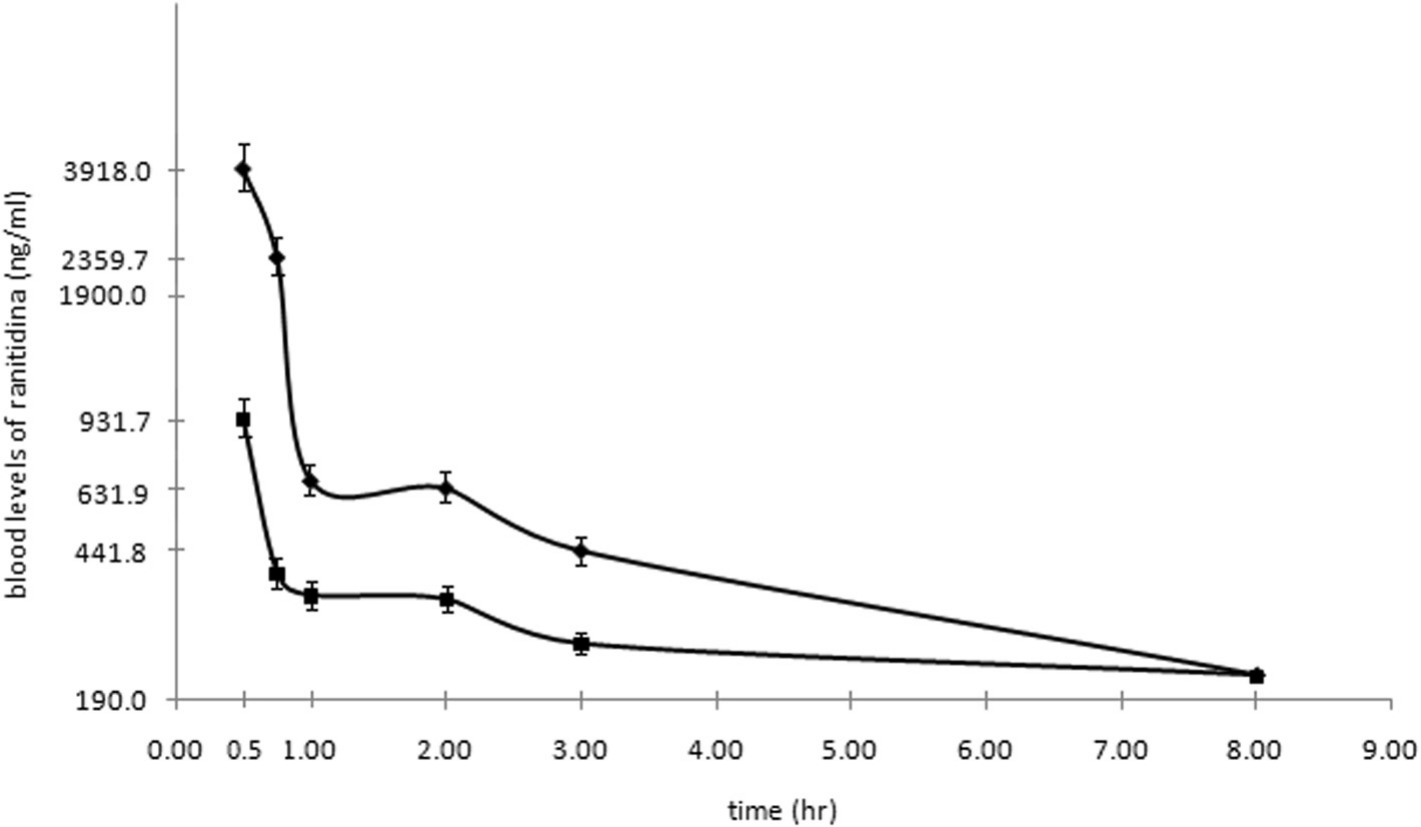
Pharmacokinetics of ranitidine in preterm (top line) and term (bottom line) neonates after intravenous dose of 3 mg/kg

The average value and standard deviation of the gestational age were 34.2 ± 4.16 weeks for female newborns and 35.8 ± 2.7 weeks for males (*p* = 0.103). However, even when the birth weights were different in the male and female newborns with average value of 2330.7 ± 677 g for girls and 2,680.5 ± 661 for boys, there was no statistically significant difference *p* = 0.715. In all the patients, there were no clinical signs or laboratory evidence of renal and/or liver dysfunctions. The pharmacokinetic profiles of the preterm as well as the term neonates are shown in Fig. 1.

Plasma concentration data were fit to a two compartment (bi-exponential) model after the applications of residual methods. The calculated pharmacokinetic parameters are reported in Table 2.

**Table 2:** Pharmacokinetic parameters after data adjustment for a model of two compartments for full population. Average ± standard deviation

AUC (Area under curve)	1688.9 ± 562.6 ng/mL/h
t½ of K_10_	1.69 ± 0.56 h
t½ α (Half life distribution)	0.3899 ± 0.129 h
t½ el (Half life elimination)	2.79 ± 0.93 h
K_10_ (Velocity constant)	0.9105 ± 0.34 h^−1^
K_12_ (Velocity of transference)	0.5395 ± 0.176 h^−1^
K_21_ (Velocity of transference)	1.76 ± 0.58 h^−1^
V_d_ (Volume of distribution)	1.44 ± 0.48 L/Kg
Cl (Clearance)	5.9 ± 1.96 mL/Kg/h

In Table 3, the median, minimum, and maximum values of plasma concentration of ranitidine of the newborns based on gestational age are shown.

**Table 3:** Median, minimum and maximum values of plasma concentrations of ranitidine (ng/ml) in newborns of both sexes, based on gestational age

Diagnoses^a^	No. of Cases	Values		
		Median	minimum	maximum
SGA Pre-term	6	518	197.66	937.6
AGA Pre-term	20	1484	61.20	7817.6
LGA Pre-term	4	1337	684.18	3687.9
SGA term	7	217	55.39	3080.7
AGA term	13	343	45.94	1075.7

^a^ Classification according to Jurado García (15)

Although, the number of cases is different considering the gestational age, the highest number of subjects corresponded to AGA pre-term newborns (20 cases), and the least to LGA pre-terms (4 cases). These two groups presented the most elevated concentration values of ranitidine at zero hour with median value of 1484 ng/mL and 1337 ng/mL respectively.

In the case of intravenous single dose, the plasma concentration of ranitidine was ≥ 400 ng/mL in 34 (68 %) of the newborns. Concentrations within the normal therapeutic range (100–400 ng/mL) were seen in 11 (22 %) of the neonates while 5 (10 %) had concentrations below sub-therapeutic range (<100 ng/mL). The analysis showed statistically significant differences with *X*^2^ = 28.12 and p ≤ 0.01. There is a wide variation in the plasma concentration of ranitidine which decreases as the gestational age increases. This explains the variability in ranitidine plasma concentration in early gestational age.

Within the first 30 min, the pH value of all the patients was ≤ 4. There was an increase of > 5, with the intragastric pH being > 4 in a minimum of 15 h, which was registered during the study by gastric aspirates.

## Discussion

Ranitidine is a drug used in the treatment of digestive tract bleeding, primarily in the upper segment, due to mucus alteration during neonatal stage. In addition, it is highly used in the prevention of gastric bleeding in neonates requiring therapeutic management in intensive care units. However, studies on PK of this drug in a risk group like newborns are very scarce. This is the basis justifying this study.

Fontana et al. [20], on studying PK values of ranitidine in term neonates reported t½_el_ values of 3.45 ± 0.31 h; V_d,_ of 1.52 ± 0.91 L/kg; and Cl of 5.02 ± 0.46 mL/kg/h. Wiest et al. [21], on their part reported t½_el_ values of 2.09 h; V_d,_ of 1.61 L/kg; and Cl of 13.9 mL/kg/h in neonates from 2 to 21 months of age. A comparison of V_d_ values in both studies with 1.6 L/kg reported in adults by Schaiquevich et al., [22], reveals that plasma protein binding and tissue distributions of this drug are similar in different age groups.

The value of t½_el_ in the study of Fontana et al. [20], was more prolonged than that reported by Wiest et al. [21] (3.45 vs 2.09 h). Consequently, Cl was less (5.02 vs 13.9 mL/kg/h). In a study carried out by Mallet et al. [4], t½_el_ value of 2.8 h was obtained in neonates of 6 weeks to 6 months old while in other studies made in older neonates by Zhang et al., [23] and in adult by Schaiquevich et al., [22], t½_el_ values of 1.8 and 1.9 h respectively were reported. These evidentially depict that the most prolonged t½_el_ value reported in newborns precisely reflects the lowest glomerular filtration velocity different from what is observed in older neonates where t½_el_ values were found to increase in the first three weeks of neonatal life [24].

The use of ranitidine with other drugs in a disease such as gastro-esophageal reflux is common. Gastro-esophageal reflux is a common sickness of upper gastrointestinal motility that widely differs in severity and prognoses. The knowledge of ranitidine pharmacokinetics and other drugs that are usually combined with it for the treatment of this pathology is important, this would help to optimize the therapeutic benefits. Patients with gastro-esophageal reflux are usually elderly people and the pharmacokinetic variability in this group of population is evident. Moreover, the gastro-esophageal reflux, the basal pathology, is usually accompanied by symptoms and other pathologies that require medical management with other compounds. The ideal therapy for esophageal reflux should have a linear pharmacokinetic and a relatively longer plasma half-life (t½_el_), a duration that would permit its administration once a day, as well as a stable effect independent of its interactions with food, antacids and other drugs. In the present study, total clearance of ranitidine was well correlated with glomerular filtration velocity. An administration of ranitidine (3 mg/kg/24 h) for 72 h was reduced for 24 h, suggesting that in full-term newborns with stable hepatic and liver functions, the administration of ranitidine needs not be more frequent than 12 h, and that the treatment response must be monitored with repeated measurement of gastric pH and the doses adjusted in conformity with the response obtained.

This study would permit the determination and identification of ranitidine concentrations in newborn patients. Kuusela, [25] studied critically ill pre-term and full-term newborns to determine the optimum ranitidine doses through gastric pH monitoring. The same was investigated by Fontana et al. [12]. Their results showed that critically ill newborns were able to secrete intraluminal gastric acid, and that ranitidine concentrations correlated with gastric pH values ≥ 4. They concluded that a significantly lower dose of ranitidine is needed in pre-term compared to full-term newborns in order to maintain their intraluminal gastric pH over 4.

The prevailing adjusted doses on prescription of this drug for newborn patients have the disadvantage of either sub-therapeutic concentration levels or concentration levels with high risk of overdosing [26]. Therefore, the identification of these concentrations is of paramount importance if we want to get the maximum therapeutic benefit. The results of the present study suggest a modification in the treatment regimens in Neonatology service of General Hospital of Durango, Mexico, as was recommended in previous studies [27, 28].

## Conclusions

Plasma levels of ranitidine depend on the gestational age of a newborn. At the same time, the results could contribute to a rational management of ranitidine.

It is inferred the possible relationship between after-birth age and pharmacokinetics of the neonates as their internal organs get matured without minding their gestational background.

## Abbreviations

AGA, appropriate for gestational age; AUC, area under curve; Cl, clearance; CV, coefficient of variation; GA, gestational age; h, hour; HPLC, high performance liquid chromatography; LGA, large for gestational age; mcL, microliter; mL/min, milliliter per minute; NaOH, sodium hydroxide; NICU, neonatal Intensive care unit; PK, pharmacokinetics; SGA, small for gestational age; t½_el_, half-life elimination; t½β, bi-exponential half-life elimination; UV, ultraviolet; v/v, volume/volume; Vd, volume of distribution; μm, micrometer.
